# Music Listening as Exploratory Behavior: From Dispositional Reactions to Epistemic Interactions with the Sonic World

**DOI:** 10.3390/bs14090825

**Published:** 2024-09-16

**Authors:** Mark Reybrouck, Piotr Podlipniak, David Welch

**Affiliations:** 1 Musicology Research Group, Faculty of Arts, KU Leuven-University of Leuven, 3000 Leuven, Belgium; 2 Institute for Psychoacoustics and Electronic Music (IPEM), Department of Art History, Musicology and Theatre Studies, 9000 Ghent, Belgium; 3 Institute of Musicology, Adam Mickiewicz University in Poznań, 61-712 Poznań, Poland; podlip@poczta.onet.pl; 4 Institute Audiology Section, School of Population Health, University of Auckland, Auckland 2011, New Zealand; d.welch@auckland.ac.nz

**Keywords:** exploratory behavior, coping, cognitive load, prediction, active sampling, curiosity, defamiliarization

## Abstract

Listening to music can span a continuum from passive consumption to active exploration, relying on processes of coping with the sounds as well as higher-level processes of sense-making. Revolving around the major questions of “what” and “how” to explore, this paper takes a naturalistic stance toward music listening, providing tools to objectively describe the underlying mechanisms of musical sense-making by weakening the distinction between music and non-music. Starting from a non-exclusionary conception of “coping” with the sounds, it stresses the exploratory approach of treating music as a sound environment to be discovered by an attentive listener. Exploratory listening, in this view, is an open-minded and active process, not dependent on simply recalling pre-existing knowledge or information that reduces cognitive processing efforts but having a high cognitive load due to the need for highly focused attention and perceptual readiness. Music, explored in this way, is valued for its complexity, surprisingness, novelty, incongruity, puzzlingness, and patterns, relying on processes of selection, differentiation, discrimination, and identification.

## 1. Introduction

Music has inductive power. It can move people to tears by triggering a cascade of neurochemical reactions in the body. These reactions have been studied in the context of the neuroendocrinology of musical reward, as part of the biochemistry of emotions and affect [1,2] with a major focus on the working of the dopaminergic reward system [3,4,5,6,7,8]. These neurochemical reactions, however, are not always automatically triggered in listeners, who may rely on distinct listening strategies, some of which could be rudimentary or highly sophisticated. The same music, therefore, can elicit different reactions among respective listeners because each listener’s level of sophistication may govern the ways that music can influence them. This means that music listeners should not be considered merely as passive recipients triggered by the acoustic features of the sounds. Skilled listening, to the contrary, should entail a sequence of states that are induced by active engagement with the music and rely on processes of coping with the sounds [9] and higher-level processes of sense-making [10].

**Figure 1:**
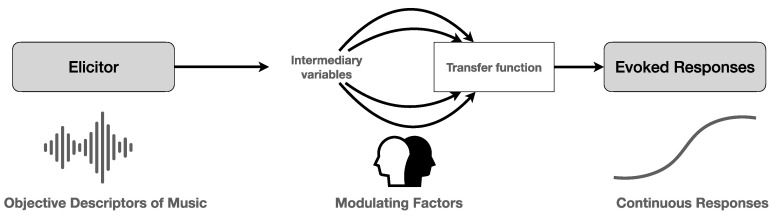
Overview of the elicitor–response schematic with modulating factors and transfer function. Reprinted from [11]. (Copyright © 2022 Reybrouck and Eerola. Creative Commons Attribution License (CC BY)).

In this paper, we elaborate on this active engagement, starting from the conception that music listening is a time-oriented phenomenon of exploratory activity that brings together perception, emotional–affective resonance, and cognitive explorations of the music. We see two broad issues to be addressed here: “what” is to be explored? and “how” is it to be explored? To answer them, we propose to distinguish between three descriptive moments of musical information gathering: (i) the description of music from a third-person perspective, relying on an objective description of the acoustic features of the music as an eliciting stimulus; (ii) a description of possible mediating factors between the eliciting stimuli and the responses by the listener; and (iii) a description of the continuous and real-time physiological and cognitive–affective responses by the listener. Figure 1 provides a depiction of these major moments in terms of an elicitor–response schematic, somewhat analogous to a mathematical function with as the domain (the input), all possible acoustic features, as the range (the output), all possible responses, and as mapping between the input and the output, a transfer function that embraces a whole bunch of mediating and modulating variables [11]. A lot of research has been conducted already on the acoustic description of the music—see, e.g., the set of acoustic features that capture the timbral, rhythmical, and tonal properties of music as operationalized in the MIRtoolbox [12,13]—and on the evoked responses to music [14,15]. In this paper, however, we focus mainly on the mediating variables which may function as modulatory factors. It should be noted also that the arrows in the figure do not necessarily stand for a linear and unidirectional succession, as the responses can also influence the modulating factors. However, the logical distinction between the three major moments has a lot of descriptive and explanatory power.

In what follows, we first elaborate briefly on the objective description of the sounds, relying heavily on what has been coined the naturalization of musical epistemology. We then dig deeper into the distinction between sound and music by considering the mediating role of exploration, active sampling, and curiosity. We thereafter expand on the combined cognitive–affective reactions to the music. We then bring all of this together in an ecological model of coping with the sounds, which conceives of music as an affordance-laden structure that has the potential of being interpreted by the listener. As the latter has pedagogical and clinical implications, we end our overview by arguing for a conception of exploratory listening as a learnable skill.

Given that our review paper brings together several scientific discourses in an interdisciplinary way, it may be difficult to see the coherent picture. We therefore present in Box 1 a short list of core messages that may be helpful to grasp the development of our arguments.

Box 1 **Overview of some core messages of the arguments developed throughout the paper.**

**CORE ASSUMPTIONS**

- Listening may span a continuum from passive consumption to active exploration.
- Active exploration is a way of coping with the sounds.
- Coping relies on behavioral strategies of curiosity and active sampling of unexplored phenomena in the environment.
- Coping as a broad category blurs the distinction between music and non-music and, in certain circumstances, values music as a sound environment.
- Exploratory listening implies a high cognitive load by heightening focal attention and perceptual readiness.
- Exploration is not equated simply with coping behavior; it also relies on prediction, expectation, and valuation.
- The aesthetic experience implies an exploratory attitude and enjoyment of active exploration.

## 2. The Objective Description as a Starting Point: Naturalizing Musical Epistemology

The distinction between the above-mentioned three levels of description elucidates the mechanisms of musical sense-making and interactions with the sounds. It is an approach that is related to the naturalization of musical epistemology, as advanced already in the *naturalization of aesthetics*, which studies the aesthetic experience from a biological and adaptive perspective. Relying heavily on neuroscience and evolutionary biology, it challenges to some extent the distinction between art and non-art [9,16], allowing a conception of music in terms of sounds, which can be studied for their own sake but also for their eliciting power. As such, there are lots of opportunities for measurement and empirical investigation both with respect to the stimuli and the responses, but also for the mediating variables that may modulate the linearity and causality of the input–output mapping. Care should be taken, however, to avoid the pitfalls of reductionism by using the “divide and conquer” method of research, which may inappropriately ignore variables due to the principle of parsimony [17].

Using such a reductive explanatory principle may prove useful to investigate phenomena however. This holds in particular for laboratory conditions, which aim at inferring causality through a properly designed experiment to demonstrate that A has affected B rather than B having affected A. Such causality cannot be demonstrated if it is not possible to control a situation without disrupting the interactions, as in the real world where controlling aspects of the environment would be unrealistic, and interpretations rely on correlation rather than on causation. It makes sense, therefore, to argue for a combined approach that brings together the methodologies of the hard sciences, with their emphasis on measurement and quantification, and the humanities, with their reliance on psychological, cognitive, social, and cultural variables. It is an approach that is applauded in the field of *cognitive humanities* as an emerging interdisciplinary field that brings together the use of experimental cognitive methods on the one hand, and the development and critical reflection of concepts, models, and approaches on the other hand [18].

The naturalistic approach is an interesting starting point in this regard. It provides some tools to describe in objective terms some of the underlying mechanisms of musical sense-making and interactions with the sounds. In addition, it avoids discussions about the thin dividing line between music and non-music by referring to music as vibrational and transferable energy that impinges on our body and our senses. This process can be described at distinct levels of responding, with a dynamic tension between lower-level reactive processes and higher-level affective and cognitive functioning. It may be construed as “coping with the sounds” [9], which, as a non-exclusionary term, can be applied to all kinds of sounds that trigger a reaction. It is a conception that considers music as a sound environment, with the possibility of looking for both familiar and unknown elements. This distinction may seem trivial at first sight, but it is important as it refers to the difference between *data-rich* and *data-poor* fields of study, which are characterized by the volume of pertinent evidence. As Huron states: “When the evidence is minimal, researchers in data-rich fields have the luxury of suspending judgment until more evidence is assembled. By contrast, researchers in data-poor fields often must interpret a set of data that is both very small and final—with no hope of additional forthcoming evidence” [17] (p. 10).

Traditional musical analysis can be regarded as a data-poor approach in the sense that it is limited by the extent to which theorists’ opinions are respected and align with others’ thinking. On the other hand, thinking of music listening as *exploratory behavior* allows a more data-rich approach. It is an approach that conceives of the sounding world as an enchanting place, with the possibility of collecting newly generated evidence for what listeners may consider to be meaningful. This evidence can be gathered in a natural way in the case that listeners use their dispositional tools for observation, but this natural method of data gathering can also be enriched by the opportunities and tools of modern empirical science, such as computational and database resources, sophisticated modeling techniques, and other innovations “to collect, analyze and interpret musically-pertinent evidence and artifacts” [17] (p. 25). It is an interesting broadening of scope that encompasses the study of the acoustic structure of music, its symbolic representations, and the broader field of musical sense-making that relies on dispositional tools for coping with the sounds with subdomains of perception, emotion, affect, and cognition [19]. Together, these domains should provide a fuller engagement with music as an affordance-laden structure [20] that triggers listeners to be more receptive.

The exploratory perspective of valuing music as a sound environment, further, can benefit from current and previous research in the fields of experimental aesthetics—with a focus on feelings of liking, preference, pleasure, and beauty [21,22,23]—the psychological study of emotions [24,25], and peak experiences or unusual states of consciousness, such as aesthetic chills, being touched or being moved by the music [26,27,28]. All this is firmly grounded in empirical research, which makes it possible to investigate the transformation from an ordinary sensory experience to a full-fledged musical–aesthetic experience. Two major components of affective processing are important here: *arousal* and *valence*, which, together, are constitutive of the empowering impact of music as an eliciting stimulus [29,30]. They seem to function as significant explanatory variables to explain how listeners cope with sounds in terms of pleasure, displeasure, activation, and arousal and make it possible to describe the process of exploration in terms of biochemistry and physiological and neural correlates [15].

## 3. From Sound to Experience: The Mediating Role of Exploration, Active Sampling, and Curiosity

The exploratory approach to music listening celebrates an experiential approach to musical sense-making with two major implications: music is an acoustical structure that evolves over time and listening is an exploratory activity that keeps track with the unfolding over time. In this context, it is possible to incorporate the fine-grained minutiae of the sonorous articulation. The approach values the lived experience of music, echoing somewhat the experiential legacy of pragmatic thinkers such as Dewey and James (see [31] for an overview).

### 3.1. The Pragmatic Legacy: Experience and Enaction

The founders of pragmatic philosophy have highlighted the major role of experience. Dewey, in particular, has pointed repeatedly to the importance of *having an experience*, with our senses figuring as sentinels for monitoring the external environment to be prepared for future action and unexpected events [32]. He pointed out, moreover, that an artistic experience is not basically different from a natural experience, with differences in degree rather than in quality. This could mean that listeners should build their listening strategies on natural strategies of perception, foreshadowing somewhat the *sentinel hypothesis* in neuroscience [33,34,35], which states that the default network of the brain plays a major role in broadly monitoring the external environment in a kind of exploratory state [36] or state of watchfulness [37]. This default approach, however, is just a starting point. The transition from naïve to expert listening, therefore, should go beyond passive registration of incoming signals, by learning to invest in active processes that are characterized by exploring, selecting, modifying, and focusing of attention, all of which have already been the subject of previous theorizing on perceptual learning and development (for an exhaustive overview, see [38,39,40]). James, on the other hand, has launched his less-known epistemological doctrine of *radical empiricism*, which revolves around the major role of knowledge-by-acquaintance as the kind of knowledge of things that is generated by their presentation to the senses. Distinguishing between “concepts” and “percepts”, he has argued for combining conceptual knowledge with perceptual reality. Conceptual knowledge, in his view, remains superficial and abstract and is thus inadequate to address the fullness of reality to be known [41], [42] (p. 327).

There is actually renewed interest for the legacy of this first generation of American pragmatists, who have been considered also as the first cognitive scientists. Their insights have been very influential for the promotion of a naturalist approach to experience in general, and their emphasis on explanation, justification, and scientific methodology can be seen as a foreshadowing of the emerging field of *neuropragmatics* [43,44]. Dewey’s theory of knowledge [45,46] claims a functional separation between observed and experienced phenomena on the one hand and theoretical constructs on the other. His epistemological claims criticize the narrow and one-sided “spectator theory of knowledge”—understanding knowledge as apprehension of fixed and independent objects on the part of the cognizing subject—and align strongly with *operationalism*, in the sense that the objects of knowledge are not given in an external and detached way but are constituted operationally by inquiry in a particular contextual whole (see [47] for an overview). Constructivist learning theories, therefore, have advocated the hypothesis that learners learn best when they must discover or construct essential information for themselves, rather than being merely presented with it as in the case of guided instruction (see e.g., [48,49,50]). Proponents of minimal guidance during instruction have pointed to the benefits of *discovery learning* [48], with strong grounding in *constructivism*, which claims that knowledge is constructed by learners when presented with goals and minimal information [51]. Knowledge, in this view, can best be learned through experience as advocated by various theories of learning and/or teaching, such as discovery learning, problem-based learning, inquiry-based learning, experiential learning, the competency-based approach, situated cognition, and constructivist learning, including radical constructivist and social constructivism (see [52] for an overview). The common feature of these approaches is that individuals construct their knowledge through experience, which has the advantage of an “unbiased observation” as in the best inductive and empiricist tradition. They emphasize direct experience and individual inquiry, which together can be subsumed under the concept of discovery [53].

The discovery approach has been criticized however. There are, first, the criticisms raised against Dewey’s epistemological theory of knowledge, which, taking a naturalistic interpretation of human cognitive development, may overestimate the role of practical and empirical work to the detriment of the equally important role of theoretical mastery [47]. Other concerns are that an exclusive focus on discovery learning puts too much stress on working memory instead of relying on the benefits of long-term memory as a central, dominant structure of human cognitive architecture. Long-term memory is not merely a passive repertory of isolated fragments of information to be recalled but an aid to quickly select and apply the best procedures to solve a problem. It makes people skillful in an area because it contains huge amounts of concerned information [52,54]. Bypassing to some extent this available information puts greater strain on working memory, which is especially effective in processing new information but which is limited in duration and capacity. Processing information, moreover, requires more demanding efforts than merely storing or recalling it, as evidenced by the *cognitive load theory* [55,56,57], which claims that free exploration of a highly complex environment may generate a load on working memory that may limit learning [58,59].

Listening to music is an interesting case in this regard, in the sense that the cognitive load of perceptually tracking the sonorous articulation of music or a sound environment should not necessarily be seen as a limitation, but rather as an advantage. A full-fledged musical–aesthetic experience, in fact, does not aim at lowering processing efforts. It calls forth, on the contrary, a heightening of focal attention and perceptual readiness, with openness to experience being more important than recalling or recognizing crystallized schemes of knowledge that lack the particularities of a real-time perceptual experience [60]. Consideration should be given, therefore, to the model of humans’ cognitive architecture that includes both a permanent knowledge base in long-term memory and temporary conscious processing in working memory that itself can be integrated with long-term memory through the learning process. Cognitive load theory recognizes these structures and their interplay in explaining how humans interact with information to be learned.

The distinction between *intrinsic*, *extraneous*, and *germane* cognitive load is central to this (see [61] for an overview). Intrinsic load is caused by the internal complexity and degree of interconnectedness between those elements of information that should be simultaneously held in working memory versus that which is already available in long-term memory. It depends on the learning task and on the level of expertise of the learner. Extraneous cognitive load, on the other hand, is not dependent on cognitive processes that are not necessary for achieving goals but depends on the way learning tasks are organized and presented. As such, they can increase unnecessary demands on working memory, as in split-attention situations. Germane cognitive load, finally, refers to the cognitive load needed to understand new ideas and recognizes that no learning can occur without effortful cognitive and related working memory load [62,63].

It is not possible to sharply distinguish intrinsic from germane cognitive load, as learning anything complex causes both. They can be seen as a “good” cognitive load that directly contributes to knowledge acquisition in contrast to extraneous cognitive load, which is considered a “bad” cognitive load in that it is a result of poorly designed learning approaches [61]. What really matters is the process of establishing connections between new elements in working memory and knowledge structures in long-term memory.

The perspective of cognitive load theory aligns with the pragmatic emphasis on having a full-fledged perceptual experience in that heightened cognitive load may be a beneficial effect of the process, which ultimately leads to deeper understanding and cognitive growth. This view has also received support from recent paradigm shifts in cognitive science, with growing criticism of disembodied and symbolic-computational approaches to cognition in favor of an embodied and enactive approach [10]. The latter must be seen as a reaction against a reductionist approach to cognition that elucidated very little about what it means to be human in everyday, lived situations [64] (p. xv). Enactive frameworks, therefore, have tried to bring experience and cognition to the forefront of cognitive science, stressing the centrality of the phenomenology of an organism’s experience of the world and of itself. It is a conception that radically rejects the idea of the mind as a mirror of the external world [65], or, as the founding enactivists have put it: “cognition is not the representation of a pregiven world by a pregiven mind but is rather the enactment of a world and a mind on the basis of a history of the variety of actions that a being in the world performs” [64] (p. 9).

### 3.2. Experience as Exploratory Behavior

Listening includes sensory experience. It is linked to the hearing system, which evolved to detect acoustic energy changes in the environment and to interpret acoustic signals and cues in terms of optimal navigation in this environment [66]. There are multiple ways to interpret the environment around us, but the starting point is the primordial corporeal–sensory openness of an organism to the world. This provides the basis for perception, consciousness, and intentionality and allows human beings to enact a world of meaningful things and relationships, resulting in the construction of reliable interactions with their environment. Such reliability is important for survival and well-being as it affords the interpretation of meaning from cues and signals, and it also entails the ability to adapt to contingent changes and to enact new perceptual relationships with the world. Applied to music, this should mean that an individual’s musical growth and development should depend on the ability to find a balance between reliability and novelty [67] (p. 157).

Music listening, accordingly, can be considered as exploratory behavior, with listeners being invited to manage their attentional focus and levels of processing in search of meaningful carriers of meaning. Music, in this view, can be conceived as a sonic environment, which can be surveyed in terms of complexity, surprise, novelty, incongruity, puzzlingness, and patterns, relying on processes of selection, differentiation, discrimination, and identification, somewhat analogous to the way that organisms “cope” with their surroundings [68,69]. This implies ways of listening that balance between the search for safety on the one hand, and the search for novelty and challenges on the other [70,71]. Such an exploratory dynamic implies the possibility of gathering information from an environment that is complex enough to be promising but not so complex as to be unreadable [66]. It is an approach that echoes somewhat Berlyne’s psychobiological contributions to empirical aesthetics, with a major claim that aesthetic appreciation can be defined as a function of perceived arousal [21]. This can be seen as an extension of the inverted U-shaped relationship between stimulus intensity and pleasure—the co-called Wundt curve—which means, basically, that a stimulus’ liking is linked up to a certain level of intensity, but when the intensity exceeds a critical threshold, pleasure may decrease [72]. This basic claim has been extended to the field of discrimination learning under the influence of task difficulty and stress and has become known as the Yerkes–Dodson law, which states that the optimum motivation for a learning task decreases with increasing difficulty [73] (see also [74]). The law has provoked a lot of heated debate and had a major impact on cognitive research with decades of both support for and criticisms of its heuristic value (see [75] for an overview).

It is tempting to translate this to the realm of music. Each musical stimulus, in fact, has arousal potential, which refers to the excitement of the nervous system in response to a stimulus. Optimal arousal is likely to be afforded by stimuli that are situated within the optimal zone of stimulation. There is a danger, however, of generalizing too much by claiming linear or non-linear relationships between arousal and general responsiveness without considering mediating and modulating factors such as individual learning histories, personality traits, and current levels of competence. Additionally, because music is typically a multifaceted stimulus composed of interwoven levels of structure such as rhythm, meter, melody, harmony, and timbre, multiple coexisting optimal zones of stimulation for each structural level must be considered. It makes sense, therefore, to also try to explain the induction of arousal in terms of information processing, with a dynamic tension between *originality* and *banality* [76,77], or, in other terms, between *entropy* and *order* [78]. This distinction seems to have a lot of operational power in the sense that arousal can be modulated by variables such as complexity, surprise, ambiguity, and novelty. These variables, however, are not constants, which means that they can vary over the course of a lifetime and among individual listeners. What is complex for one listener, may seem banal to someone else, and surprise is no longer surprising after repeated listening. The field of music, therefore, is quite challenging, and this all the more so in the case of non-familiar music, such as, e.g., atonal music or music from foreign cultures, which, when experienced as being outside of the listener’s comfort zone, may have a high arousal potential [79]. Music, in that case, can be valued as a high-uncertainty environment that engages neural and cognitive mechanisms that are associated with exploratory behavior, and that can fuel a cascade of responses. The latter can be experienced as frightening and threatening but they may be helpful also in considering predictive processes and hedonic value that underlie the reception of music. It can be argued, moreover, that such exploratory behavior is a crucial facet of an aesthetic experience in general.

One of the explanatory mechanisms, in this regard, is the reliance on *predictive models.* Such predictive dynamics are related to the generation of expectations about future events, or, in the case of music, about how the music will evolve, with a dynamic interplay between violation and confirmation of these expectations. This is quite unproblematic in the case of predictable music, such as tonal music [80,81,82], but for more challenging music, there is a need to consider more closely the interplay between bottom-up and top-down expectations. The former can be related to Gestalt principles of bottom-up grouping with respect to pitch and temporal proximity [83], rhythmic grouping [84], and sound similarity [85,86]. The latter are modulated by top-down mechanisms driven by the perceptual impulse to find regularities in the environment [87,88,89,90].

The *predictive coding theory* has a lot of explanatory power in this regard (see [91,92] for an overview). It states that living organisms must minimize *free energy*, which refers to the divergence between observed and expected data, given an agent’s generative model. Sensory input that is inconsistent with the agent’s generative model propagates prediction errors in order to change the generative model and allowing the organism to sample the environment in such a way that this maximizes evidence for the generative model [93,94]. It is a mechanism of active inference that allows an agent to use its model to infer the most likely causes of observable outcome [95].

Combining both bottom-up and top-down processes allows the brain to function as a prediction machine that continuously predicts future sensory input by means of higher-level perceptual models. Top-down predictions are matched with bottom-up sensory input, and if they do not match, the “prediction error” causes the model to update in line with the sensory representation of the external reality [89,96]. This can be measured objectively through the mismatch negativity (MMN), which is a variation in the brain’s electromagnetic response to sounds that deviate from predictions in a regular sound environment (see [97,98] for auditory applications). The predictive models adapt to the statistics of the incoming stimuli, to become weaker with increasing predictive uncertainty [92,99,100] so that in high-uncertainty conditions, predictions are greatly attenuated. This serves to reduce or even nullify the predictive errors [101,102,103]. On the other hand, in conditions such as hearing loss, where there is a dearth of sensory information to bring predictive models under control, hallucinations may be experienced as a result of the predictions dominating the perceptual environment [104].

The predictive coding framework also has explanatory power for the understanding of music enjoyment [95,105,106] in the sense that music listening is characterized by the interplay between levels of predictability or expectations and the way these unfold over time. Resolving uncertainty, therefore, is one of the elements that makes music listening challenging and rewarding, and it is typical also for states of exploration [88,107]. As described above, however, predictive models are attenuated when music—or environments in general—lack a recognizable structure and when listeners are inclined to more passively accept merely what is offered by the music [82] (p. 331). This has been demonstrated in recent empirical research using the MMN that showed a reduction in the predictions in musical sequences that had higher levels of uncertainty [108,109,110].

The relation between subjective valuing and sensory processing, however, is not yet totally clear, with an assumed dissociation between sensory and cognitive musical expectations [79]. It can be questioned, in this regard, which experiential dimensions underlie the aesthetic experience in the case of high-uncertainty musical predictions, and which hedonic values, appreciations, and pleasurable experiences can be demonstrated. A recent study showed a major distinction between expert listeners, with a specialization in atonal music, and listeners with a preference for classic-romantic music [111]. The former reported an adoption of their exploratory attitude as well as enjoyment of the active exploration of the music, together with the possibility to continuously seek new ways of engaging with the music and to probe these ways through listening. They also mentioned the experience of pleasurable moments that emerged from the recognition of an underlying pattern or structure in the music. Such “joy of discovery” accompanied the moments of perceptual insight, and was experienced as an increase in coherence and confirmation of self-generated expectations. They reported also their adoption of an open stance together with an emergent feeling of curiosity, not as a “trait” level but as a “state” of curiosity and openness during engagement with the music. Not much research has yet been done, however, on how the perception of change while listening can lead to an increase in the feeling of curiosity [112,113]. Generalizing a little, it seems that the feeling of discovering a pattern, which makes it possible to encounter a predictable event in a high-entropy context, may bring engaged listeners to feel greater curiosity and an inclination to explore the sounding music as it unfolds, suggesting again the search for coherence and decrease in complexity. The analogy of watching flames or clouds that, in their chaotic motion, seem sometimes to form patterns or even recognizable objects, may be illustrative of this tendency. This search for pattern discovery, moreover, holds, in particular, in contexts that seem to be less structured and predictable, which means that low-predictable contexts afford listeners an exploratory attitude [79,114]. Exploratory behavior, therefore, is essential in novel and complex environments with surprising elements [115], corroborating the idea that humans intrinsically seek knowledge [116], starting from a drive for curiosity [117] and an active engagement with new environments [118] with the goal to reduce uncertainty.

### 3.3. Active Sampling and Curiosity

*Exploration* is directed at learning about the properties of an uncertain environment [119] in contrast to *exploitation*, which values benefitting from a familiar environment with known and obtainable rewards [118]. Several mechanisms related to *active sampling* and *curiosity* [118,120,121] seem to qualify in this regard. Given the abundance of potential information in the environment, intelligent beings may be inclined to sparsely sample the rich incoming sensory information, reflecting the protective mechanism for organisms with limited processing capacity that can “sense” more information than they can “process”.

The question can be raised, however, as to which motives drive attention and curiosity so that some sources of information become more attention-worthy than others [122,123]. A possible answer can be found in the commonalities between attention, curiosity, and decision making as highlighted in the *exploration–exploitation* literature that tries to ground the factors that motivate humans or animals to engage with specific stimuli. It is an approach that proposes strategies for reducing the momentary uncertainty to maximize long-term gains [124] with a tradeoff between the gathering of rewards from well-known, familiar options (exploitation) versus the investigation of less familiar options (exploration) to potentially enhance the reward probability on longer time scales [125,126].

A distinction should be made, further, between *information sampling* and *information search*. The former simply amounts to the gathering of information that is relevant for a specific task, thus relying mainly on previous knowledge; the latter implies exploration not primarily driven by prior knowledge of some task or goal, but putting human beings in conditions of much more uncertainty [127]. Both mechanisms entail high levels of complexity with more or less reliance on systems of belief-based utility, which means that value can be conferred to information as a good in itself, so as to explore under conditions of many alternatives and the lack of previously acquired knowledge regarding potential rewards [128]. It is thus possible for “active” samplers to determine which sources of information they want to engage with by going beyond the selection of actions from a set of preselected and clearly presented options.

The findings show that animals—and thus also humans—may seek information, even when it does not serve any obvious purpose. It is a mechanism that is closely related to *curiosity* [129,130], which can be defined as the intrinsic desire to know, with potential questions about the state of the environment or how to manipulate it [131,132]. High information, in that view, is experienced as being intrinsically motivating [117,129,133,134].

## 4. Exploration as a Cognitive–Affective Category

The aesthetic attitude relates exploratory behavior to the experience of reward [11]. As such, it is also related to coping behavior [9], not in a narrow sense of avoidance behavior toward threatening situations, but in the broader sense of enhanced attentiveness to the solicitations of the environment. This emphasizes the importance of the regulation of attention, which can be related to exploratory behavior, arousal, and seeking reward [135]. Enhanced attentiveness, in fact, can be seen as a defining characteristic of heightened affective experiences [136,137]. At the neurobiological level, this is reflected in connections between the cortex and the limbic system—the mesocorticolimbic pathway—which is part of a larger-purpose system in animals that enables them to establish adaptive behaviors that are relevant to the availability of reward within the environment [138].

### 4.1. The Principal Dimensions of Core Affect: Valence and Arousal

The seeking disposition toward the environment is part of our biological toolkit for coping with the environmental (sounding) world. It has neurochemical correlates in the sense that it is a strong elicitor of dopamine-related hedonic pleasure, even independent of the achievement of reward [3]. Examples of potential elicitors of such altered dopaminergic firing are unpredicted rewards, prediction errors, novel stimuli, physically salient stimuli, motivational and affective salience, and attention shifts related to approach behavior [139,140,141].

The hearing system, as an evolved adaptive system to recognize changes in acoustic energy in the environment, has a major role in this regard. By searching the needed acoustic cues to optimally navigate in this environment, it is first of all a very sensitive acoustic warning system that relies on the dispositional toolkit to recognize and react to sound sources in terms of survival [66,142]. Such survival-related responses are exemplified typically in behavioral reactions to sudden changes in signal intensity, as seen in the orienting response and the acoustic startle response [143,144,145,146,147]. Human beings can discriminate among hundreds of thousands of sounds that involve distinct combinations of pitch, loudness, and timbre [14].

A distinction should be made, however, between evolutionary survival reactions, as manifested in coping behavior, and the broader concept of coping that goes beyond reactions to potential threat. The basic reactive mechanism—the release of certain neurotransmitters that heighten arousal levels in general— however, is still functioning as an evolutionary relic [148] and it can even be sought after deliberately. This is the case in musical interactions that intentionally provoke an increase in arousal and activations, as exemplified typically in listening to extremely loud music [147,149,150]. Music, in that view, can be seen as a stressor that spurs the endocrine systems to release neurotransmitters and/or hormones such as cortisol, adrenaline, noradrenaline, prolactin, testosterone, corticotrophin-releasing hormones (CRH), and adrenocorticotrophic hormones (ACTH) [151,152]. *Arousal*, however, should not be related unequivocally to stress and threatening stimuli. Together with *valence*, it has been identified as one of the principal dimensions of core affect, revolving around the activation–deactivation continuum—ranging from sleep to frenetic excitement—on the one hand (activation), and the pleasure–displeasure continuum—ranging from unpleasant to pleasant—on the other (valence) [153,154]. Together, the twin notions of arousal and valence describe the extent to which stimuli are assessed both with respect to their intensity and quality, as seen typically in the domain of emotions and emotional states [30]. Of course, there is more to music than arousal and valence—we refer broadly to the whole field of the *inductive power* of music (see [155,156,157] for an overview).

Listening to music, therefore, should not be reduced to dispositional reactions to the sounds. Music is not merely an acoustic structure that impinges on the senses. It is also a phenomenon of subjective experience and valuation with musical sense-making as the outcome of perceptual, affective, and cognitive processing, and it is up to the listener to make something coherent out of the temporal succession of the sounds. The musical experience, then, can be considered as an ongoing exploratory activity in search of affordances that are present within the music. This is illustrated by what Krueger has called the world-making power of music as “a sonic world that affords possibilities for creating, organizing, and regulating listeners’ experiences, emotion regulation and social coordination” [158] (p. 7).

### 4.2. Aesthetic Emotions and Defamiliarization

Listening to music is a multifaceted experience. It includes a combination of dispositional reactions to the sound as well as higher-level emotional–affective and cognitive processing, with levels of processing that are complementary rather than opposed to each other [9,155]. This holds in particular for aesthetic music listening, which, in contrast to incidental or everyday listening, is characterized by an increased attentional focus, allowing first initial reactions to the sounds to become available for further conscious appraisal and evaluation [159,160]. This attentional focus, together with the search for enjoyment and reward, is exemplified by *aesthetic emotions*. They have been studied extensively and have been reported in the Geneva Emotional Scale (GEMS), which assigns them to nine distinct emotion categories, namely wonder, transcendence, tenderness, nostalgia, peacefulness, power, joy, tension, and sadness [161].

Aesthetic emotions have prompted a lot of empirical research with a major focus on how aesthetic responses may elicit limbic and paralimbic activations that are involved in affective processing. There is a major dividing line between attractive or aversive properties of the stimuli [162], which raises the question as to what extent aesthetic emotions are grounded in survival-related adaptive behavior. A possible answer points in the direction of defining aesthetic experiences as partly based on exploratory behavior in a changeable environmental world. What this entails is a unique manner of engagement with the sonic world to produce adaptive and flexible behavior in terms of coping with the sounds. Aesthetic experiences, in this view, are conceived as active encounters with the sonic environment, which may figure as privileged examples that facilitate an ongoing engagement that has an exploratory and creative character. Unlike other kinds of interaction, the exploratory process of aesthetic interactions seems to have its own dynamic, independent of any goal-directedness [163]. It is a stance that echoes to some extent the claims by Shklovsky—a Russian scholar of the formalist school— that one of the major aims of art is to avoid the habitual reactions to familiar objects which tend to become automatic, unconscious, and curtailed [164,165]. What is needed, on the contrary, is a process of *defamiliarization*—also called *ostranenie*, from the Russian OCTpaHeHиe—to describe the artistic technique that presents common things in an unusual or strange way so as to induce in a spectator new perspectives or ways of looking at the world. It is a way of experiencing the world that was inspired by Aristotle—as discussed in his Poetics and Rhetoric— which describes devices and styles to defamiliarize language. One of these is the use of *Energeia*, which has been translated as “actualization” or “vivification”, and which acts on language to bring something before the eyes [166,167]. Poetic language, as contrasted with prose, is best placed to achieve this goal. Ideally, it must have the character of the foreign and the surprising so as to put brakes on perception by directing the attention to the perceptual moment itself. The aim of such defamiliarization is to make the habitual strange in order to enact the experience anew in an enhanced, sensory way [168].

The artistry of a work of art, in Shklovsky’s view, is a matter of its form being perceived as artistic, as opposed to routine perceptions that tend to become automatized and integrated in an unconscious, procedural background. Automatization, which reduces the processing effort of thought, strongly compromises the perceiver’s perceptual attunement with the world [169]. Art, to the contrary, exists to give back the sensation of life, so as to make us feel things in a more vivid way, or, as Shklovsky puts it “The goal of art is to create the sensation of seeing, and not merely recognizing, things; the device of art is the ‘ostranenie’ of things and the complication of the form, which increases the duration and complexity of perception, as the process of perception is its own end in art and must be prolonged. Art is the means to live through the making of a thing” [170] (p. 80).

Defamiliarization, therefore, is a device that triggers a special way of experiencing an object. It resuscitates the feeling of encountering something for the first time and disrupts the process of “cognitive economy” by going beyond mere recognition and appealing directly to the senses. It relies on alienation and complication to make perception more difficult and to prolong its duration [164,165]. Defamiliarization, therefore, prefers “exploration” to “recognizing”, celebrating the richness and fulness of the sensory experience as a goal in itself, rather than functioning merely as a tool, and in the terminology of cognitive load theory, discussed above, it increases the intrinsic and germane load in order to improve the profundity of the perceptual experience.

## 5. Music as an Affordance-Laden Sonic Structure

Celebrating the richness of the sensory experience broadens the scope of music to include all kinds of sounds. It highlights the role of exploratory listening and allows to conceive of music in terms of a sonic environment [171,172]. The sounds, in that view, are perceived in terms of what they “afford” to the listener, and not merely in terms of their acoustic characteristics. This is a view that opens up semiotic claims of musical sense-making as studied already in the fields of ecosemiotics and biosemiotics [172], with the former examining the semiotic relationships between an organism and its environment at large [173] and the latter exploring the biological bases of the interaction of an organism with its environment [174,175,176]. Perception, in that view, is ecologically constrained, in the sense that the world is not addressed in terms of its physical description but in terms of what it affords for survival and orientation in the environment [177].

It is not difficult to apply this to the realm of music. It suffices to think of the listener as an organism and the music as an environment. Music, then, can be considered through the lens of a natural soundscape [178], which presupposes a continuity between the perception of natural environments and music, echoing somewhat the experiential claims of Dewey [32]. Having an experience, in his view, should be defined as heightened vitality, as active and alert commerce with the world, and even if there may be a qualitative distinction between music and natural sounds, the way of listening to them appeals to the same mechanisms of exploratory behavior and focused attention. Music, then, should not be seen merely as an aesthetic artifact but also as a vibrational phenomenon that can be considered as a human-made soundscape that impinges on our body and our senses in a way that natural soundscapes also do.

**Figure 2:**
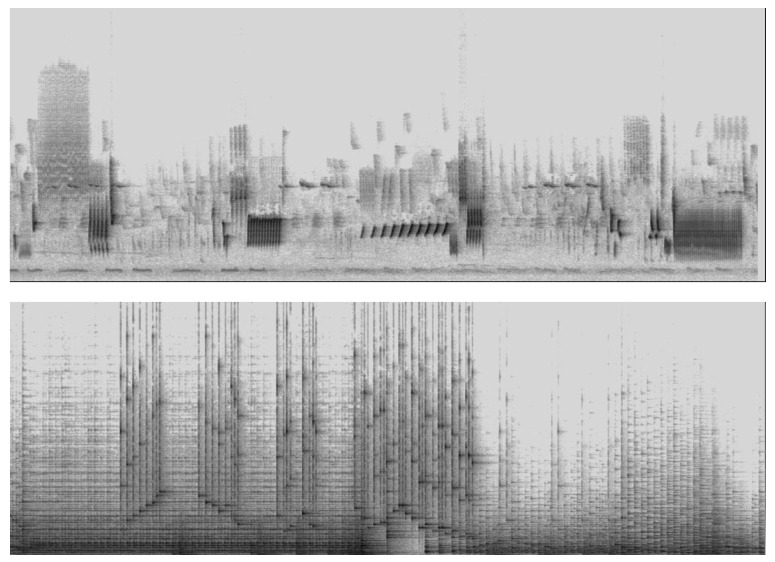
Spectrographic depiction of birdsong (nightingale, about 22 s, **upper pane**) and a piece of music (Bartók, Sonata for two pianos and percussion, 3rd movement, about 27 s, **lower pane**). The diagrams indicate time (*x*-axis) and frequency (*y*-axis) with the darker regions indicating more energy.

To make these claims somewhat more concrete, we depict in Figure 2 a spectrographic depiction of birdsong (nightingale) and a piece of music (Bartók. Sonata for 2 pianos and percussion, 3rd movement) to represent the change in spectral energy over time as the sounds unfold. As to the nightingale, what immediately stands out is the extreme richness of the spectral pattern of the song. Some listening skill is required to interpret and/or recognize the acoustic signal in such a way as to recognize the differences between the visualized acoustic patterns, and it would be challenging to listen to the song as music. Hearing and recognizing all the minutiae of the changes in its acoustic parameters, in fact, is of a different order than merely recognizing the whole structure merely as being the song of a nightingale. It is the distinction between a kind of “continuous” tracking of the sonorous unfolding and reducing this temporal course to just one “discrete” assignment of meaning to the birdsong as a whole (it is a nightingale). The spectrogram of Bartok’s piece, on the other hand, looks totally different with musical instruments being used in a rather percussive way—the depicted fragment shows the sound of two pianos, timpani, and a xylophone—but even if this music sounds very dynamic and percussive, with a richer spectrum, due to the percussive sounds, there seems to be less differentiation in the darker patterns that show louder configurations. There seem to be, as it were, significant register distinctions in exploiting the spectral space of the nightingale and Bartók’s music, and the question can be raised as to whether it is possible to differentiate between music and animal sounds on the basis of the inspection of a spectrogram. At first glimpse, there seems to be a difference in the stability of fundamental frequencies (F_o_)—or harmonics in general—over time. In the birdsong, only some F_o_’s are stable, while in the Bartók fragment, there are many more stable sound units. Moreover, the distribution of time units through time seems to be more regular in the Bartók piece than in the birdsong. This regularity allows us to feel (or experience or extract) the musical pulse, which, thanks to cognitive predictive mechanisms, constitutes a kind of scaffolding for the quantification of sounds in music [179].

The findings are challenging. They show that the distribution of acoustic energy is different in both examples. It seems that the bird is using the spectral space in a more continuous way than the music, with its reliance on more discrete categories, such as discrete pitches and simple temporal durations. The spectrographic depiction, therefore, is a most valuable tool to visualize the richness of the acoustic structure as well as its potential for inviting listeners to engage in exploratory listening. It makes it possible to listen to music as if it were a birdsong, and to the birdsong as if it were music.

Care should be taken, however, not to overestimate the role of the acoustic structure as represented by the spectrogram. Spectrographic listening—especially when this occurs in real time, with a cursor that moves along with the unfolding music—is quite appealing, but it is only a tool for exploratory listening. Such a naturalistic approach to music listening can be criticized, after all, as being reductionistic and/or ignoring the specificity of the aesthetic experience. We do not object to this specificity, but rather than conceiving of an aesthetic experience as being qualitatively distinct from the natural experience, we see it as an extension that can increase and improve the quality and the intensity of the experience. It makes sense, therefore, to start from the dispositional toolkit for coping with the sounds and to see this as the starting point for a lifelong learning curve that is grafted onto ontogenetic development. Listeners, in this view, behave as biological beings who have recourse to their neural apparatus for “coping” with the sounds, which means that they are equipped with the necessary mechanisms for evaluation of the environment in terms of threats and dangers, as well as possible benefits for survival [180,181].

Curiosity and exploitation of unexplored phenomena in the environment are important behavioral strategies in this regard. They allow animals to compete for resources that are beneficial for survival and reproduction, as exemplified most typically in the way human beings cope with the challenges of a landscape. Their responses involve the selection of places to live, the choice of habitats, emotional responses to features of the environment, positive or negative feelings of acceptance or withdrawal, exploration of the settlement, and correlations with survival and reproductive success [66].

There are, further, two distinct modes of coping with an environment. On the one hand, there is the problem-oriented, reactive survival or *coping mode*, with the aim to end or avoid potential threats and to protect viability in a deficient environment; there is, on the other hand, the *flourishing* or *co-creation mode*, which is not oriented toward threats but which aims at improving viability and the creation of better conditions for living [182]. The flourishing mode goes beyond the survival mode of basic homeostatic functioning that prompts optimal navigation in the environment in search of opportunities in the world. It entails a positive and broader redefinition of coping behavior as being highly adaptive and goal-directed by searching out those stimuli that are valued as beneficial and worthy, but also by trying to avoid those stimuli that are valued as annoying and harmful, reflecting to some extent the twin motivational systems of approach or avoidance [183].

Such a broader view on coping invites listeners—in the case of music—to actively search for stimuli that are valued as being worthy, relying on neural mechanisms for evaluating the sonic environment. These involve the management and regulation of attention and arousal with the aim to facilitate the exploratory function of “open monitoring” and to heighten the responsiveness to sensory stimulation in general [184]. This active search for potential beneficial stimuli may also lead to affective reactions and positive affect [185]. The above-mentioned “aesthetic responses” to our environment should also be seen in this context. They clearly show that humans prefer environments that facilitate and encourage exploration, wayfinding, and information gathering. Adopting an aesthetic attitude, therefore, has major implications for the “transformative power” of music, which means that even an unpleasant environment can be appreciated in an aesthetic context. This holds, for example, for the appreciation of atonal and serial music and many other musical styles in 20th and 21st century art music, which advocate a search for novelty and which sensitize the listeners’ hearing to overcome perceptual habits [186]. It is also exemplary of a change in thinking about atonal music, which has often been used rather condescendingly in neuroscientific research on perceptual and cognitive processing as an example of unpleasant and dissonant music that induces fearful emotions [187,188]. There is now another attitude that conceives of it as having a high level of predictive uncertainty [189], which opens perspectives for studying person-related factors of listeners as predictive variables for the appreciation of high-entropy music. It allows a distinction to be made between “sensory” and “conscious” enjoyment to the extent that even an unpleasant sensory experience may lead to a positive evaluation [29].

Openness to experience seems to play a major role here, together with being receptive to chills and absorption [28,190]. Music, then, can also be used for cognitive stimulation [191], which can also be aligned with the “need for cognition” [192,193] or the “knowledge instinct”, which drives people toward seeking information and discovery [117,194]. It brings together a genuine relationship between openness to experience, novelty-seeking, and preference for complexity [195].

The case of listening to music outside our comfort zone is thus an interesting one. It has the potential to change our perception, evaluation, and cognitive processing, resulting in a tolerance for surprise, disgust, and ambiguity [196], which has also been called “aesthetic framing” [197]. It means that an external context or the effect of an external situation can lead to a change in the attitude toward the music and its according value attribution [160,198], in the sense that an unpleasant sensory experience or negative emotion may be experienced differently in an aesthetic context compared to the context of a real-life situation [199]. Such an aesthetic attitude, then, is to be distinguished and detached from its utilitarian functions [200]. It thus seems that both *aesthetic framing* and *cognitive mastering* are important factors in the modulation of attitude, expectation, and appreciation [186]. They may trigger perceptual curiosity and interest to engage with music [117,201].

## 6. Exploratory Listening as a Learnable Skill

The general-purpose dispositional toolkit for coping with sounds is a major issue in music listening. It should not be seen as the only mechanism, however, but rather as a part of a broader set of abilities which are called “musicality” [202,203] and which enable the recognition and production of music. As in the case of the language faculty [204], musicality is composed of abilities that are both specific (e.g., the ability to process musical pitch, to sing, to synchronize movements with music) and not specific (e.g., the ability to recognize sound sources, to segregate sounds into separate streams) to music. The general-purpose dispositional toolkit for coping with sounds, moreover, has not evolved as the result of the adaptive value of musical behavior, though it cannot be excluded that some abilities used in music listening have served adaptive functions, such as sexual attraction [205,206,207], signaling social cohesion and strength [208,209], enhancing social bonding [210,211,212], parent–infant bonding and communication [213], deterring predators [214,215], triggering attention in parent–infant competition [209], and free-rider recognition (enjoying the benefits of a shared resource or service without bearing the fair share of the costs) [216]. Both the music-specific and non-music-specific aspects depend on learning, and this learning has its evolutionary origins in affective–emotional and cognitive functions, which are grounded in basic homeostatic regulation, with a gradual enrichment of coping mechanisms as brains grew more complex. The result is a rich interplay between bodily reactions, emotions, and cognition, with a subtle balance between survival-related behavioral reactions to sudden changes in signal intensity, and learned and acquired responses that are the outcome of an individual learning history. There is, as such, a dynamic tension between *phylogenesis* and *ontogenesis*, but the link with ancestral adaptive behavior is still looming at all stages of processing. The sounds of our recently developed, technologically advanced societies are therefore still assessed to some extent in terms of ancestral adaptive behavior.

A major question regarding the ontogenetic learning trajectory, further, concerns the role of modulating and/or mediating factors, both external and internal ones. Examples are personality traits such as empathy or openness to experience, and strategies for information gathering that make up the personal profile of each particular listener. We do not delve in this discussion here, as there is not yet conclusive evidence for the innate/acquired character of dispositional traits (see [217] for a broad overview). We want to stress, however, the major role of accumulating exposure to sounds in the course of a lifetime’s experience, which may be driven by internal or external forces. Examples are the innate curiosity, the desire to invest in exploratory behavior, and the urge to master (intrinsic motivation) or to comply with the demands of family pressure and/or educational institutions (external motivation), with cumulative effects that may result in levels of sophistication that go beyond those of mere natural maturation. Mere exposure to sounds, however, is only a first step. More important is the modulation of attention, with enhanced attentiveness being considered a defining characteristic of heightened affective experiences [136,137]. It is obvious, therefore, to relate attention regulation to exploratory behavior, arousal, and the seeking of reward [135].

Music listening, accordingly, entails the management and regulation of attention and arousal. There is, however, a major distinction between music listening and the exploration of a sonic environment. In the case of mere exploring, there is no temporal direction, which means that it is possible to direct the attentional focus at will in any order, depending only on the observer’s deliberate choice. Music, to the contrary, is a temporal art, which is characterized by the inexorability of time, with a temporal unfolding that is irreversible and even linear causal. It relies on processes of attention, memory, and expectation. Musical sense-making, therefore, can be considered as an ongoing process of knowledge construction and epistemic interactions with the sounds, with a major distinction between active, focused listening as against passive immersion in the sounds.

A difficult point in this regard is the relation between perception and attention. Attention can be understood as being partly intentional *(directed attention)*, and partly controlled by external stimuli *(captured attention)* [218] (p. 16). It is not easy to tease apart perception and attention, as attentional dynamics imply also perceptual processes [219], but this opens up new perspectives for musical sense-making by considering the listener as an active agent or acquirer of knowledge rather than a passive processor of information [220]. It highlights the role of the listener as an autonomous agent who enacts the heard music by relating epistemically and experientially to the sounds [221]. Such an approach entails the broader field of physical, physiological, and cognitive entrainment, which starts from the metaphor of *master and slave*, in the sense that listeners can behave either as follower or as leader by aligning their attentional control through small adaptations, both in terms of being driven or being the driver themselves. It allows listeners to go beyond mere reactive behavior in response to the music as an externally imposed force by taking over the role of controller, somewhat analogous to the case of dribbling a basketball. Instead of letting the ball bounce free at its own frequency, it is possible to externally impose the number of bounces, thus forcing the ball to bounce at a rate that is determined by the dribbler. The dribbler, then, is acting as a forcing function that controls the motion of the ball by imposing the bouncing rate as a driving, or forcing, frequency, which turns the free vibrations of the bouncing ball into forced vibrations. Or put differently: the ball becomes a “controlled system” with the dribbler taking the role of “controller”, provoking a continuous disturbance that controls the behavior of the system [14]. The analogy can easily be translated to the listening process by substituting the listener for the ball, and the sounding music for the dribbler. It is a beautiful example of *asymmetrical entrainment*, in the sense that the listener is forced to adjust to an external set of conditions without being able to influence them [18]. The idea, however, is at odds with the conception of the listener as an active agent. It is possible, therefore, to conceive also of a *symmetrical entrainment* with listening not being reduced to mere sensory exposure and passive resonance, but entailing a continuous and ongoing interaction with the sounds. Listeners can be entrained by the music, which acts on them in a unidirectional way. The music is then considered as an unchangeable external stimulus, which takes the role of master in the “master and slave” metaphor. It is possible, however, to challenge this unidirectional approach by emancipating the listener from a passive consumer to an active agent, who interacts in a dynamic way with the music. It is clear that explorative listening may play a major role in this regard.

## 7. Conclusions

In this paper, we have explored how distinct ways of listening may govern the way that music can influence the listener. Listening, in that view, can span a continuum from passive consumption to active exploration. The latter entails active engagement with the music, relying on processes of coping with the sounds as well as higher-level processes of sense-making. Revolving around the major questions of “what” is to be explored and “how” it is to be explored, we have taken a naturalistic stance toward the process of musical information gathering. This is an approach that describes the musical experience from a biological and adaptive perspective and that studies music in terms of sounds and their eliciting power. Such a naturalistic approach provides the tools to objectively describe the underlying mechanisms of musical sense-making by weakening the distinction between music and non-music. We therefore took as a starting point the conception of “coping with the sounds”, which, as a non-exclusionary term, applies to all kinds of sound that may trigger a reaction in one way or another. It stresses the exploratory character of valuing music as a sound environment, which can be experienced as a data-rich field, waiting to be discovered by an attentive listener. The naturalistic approach, moreover, aims at being unbiased to a great extent, emphasizing direct experience and avoiding too rapid an alignment with established categories of knowledge.

Applied to music listening, listening should not be reduced to the recalling of pre-existing knowledge or information, with the aim to reduce cognitive processing effort. What we argue for, on the contrary, is a conception of exploratory listening that celebrates a heavy cognitive load by heightening focal attention and perceptual readiness rather than relying on crystallized schemes of knowledge. Music, then, is to be valued in terms of complexity, surprise, novelty, incongruity, mystery, and patterns, relying on processes of selection, differentiation, discrimination, and identification, somewhat analogous to the way listeners “cope” with their (sonic) environment. Listening, in that view, may balance the search for safety on the one hand, and novelty and challenges on the other hand, or, in more information-theoretic terms, between order and entropy.

Coping, as a survival mechanism of living organisms in their interaction with the environment, has been defined as the “cognitive and behavioral efforts to manage specific external and/or internal demands that are appraised as taxing or exceeding the resources of the person” [222] (p. 141). It can be considered in a narrow or a broader sense, which means that it should not be restricted merely to reactive behavior to potential threats. It may also involve sense-making of the environment, including affective and cognitive interpretive behavior. The role of exploratory behavior in a changeable environment as well as the perception of environmental opportunities in this environment are of paramount importance here. Coping behavior, therefore, can broaden our behavioral and cognitive repertoire, as claimed in the “broaden-and-build theory”, which states that positive emotions consolidate and expand our resources by attempting more creative courses of action. Such an expansion may broaden the scope of attention, enhance thought–action repertoires, augment holistic processing, and increase openness to new experiences, all of which aim at building long-term resources such as resilience and curiosity [223,224,225].

Coping, however, is not all there is to exploration. It is equally important to use prediction and expectation, with possible relations between levels of uncertainty and subjective valuation of hedonic values. Much is to be expected, in this regard, from the theory of *predictive coding*, which conceives of the brain as a prediction machine by continuously predicting bottom-up sensory input based on generative top-down predictions at a higher level [87,89] with the aim to update the available cognitive model [226].

This holds in particular for an aesthetic experience, which, as a rule, depends on an exploratory attitude and an enjoyment of active exploration. In other words, besides coping, processes of active sampling and curiosity are central to music listening. Exploration, in that view, can be considered as a cognitive–affective process that brings together the regulation of attention, the experience of arousal, and the search for enjoyment and reward. Exploratory listening, therefore, is not merely survival-related behavior that is fueled by our dispositional toolkit for coping with the sounds. It is also a learnable skill that enables the listener to be an active agent or acquirer of knowledge rather than being a passive consumer of sonic information. It entails some processing effort that raises the cognitive load while perceptually tracking the sonorous articulation over time.

## Data Availability

Not applicable.
